# Physiological Auricular and Cornual Asymmetries of the Sanmartinero Creole Bovine

**DOI:** 10.3390/ani14152261

**Published:** 2024-08-04

**Authors:** Arcesio Salamanca-Carreño, Pere Miquel Parés-Casanova, Mauricio Vélez-Terranova, Germán Martínez-Correal

**Affiliations:** 1 Facultad de Medicina Veterinaria y Zootecnia, Universidad Cooperativa de Colombia, Villavicencio 50001, Colombia; 2 Department de Bromatologia, Universitat Oberta de Catalunya, 08018 Barcelona, Spain; 3 Facultad de Ciencias Agropecuarias, Universidad Nacional de Colombia sede Palmira, Palmira 763531, Colombia; 4 Asociación de Criadores de Bovinos de Razas Criollas y Colombianas de los Llanos Orientales, Villavicencio 50001, Colombia

**Keywords:** biological development, domestic animals, head, morphology, ungulates

## Abstract

**Simple Summary:**

The Sanmartinero creole bovine (SM) is found in the department of Meta (Colombian Orinoquia) and derives from the bullfighting cattle introduced by the Spanish in the 15th century. Its adaptation process has allowed it to develop adaptive characteristics to live in extreme climatic environments. Although there are studies on its reproductive and productive characteristics, little is known about possible asymmetries in some biological structures. Fluctuating asymmetry allows us to detect a possible developmental instability that may be caused by stressful conditions. The aim of our study was to present the current state of developmental instability in two bilateral cephalic characters of the SM creole bovine. The results showed a fluctuating asymmetry biased to the left for ear and horn length, and to the right for ear width and horn perimeter. We consider that the environment in which it lives may have an impact on the development of cephalic structures, creating stress in the animal, which causes the detected asymmetry. However, the authors are not aware of any studies on this topic.

**Abstract:**

Asymmetric studies can indicate disturbances in the developmental process. Fluctuating asymmetry (FA) is considered an indicator of stress. The Sanmartinero (SM) creole bovine is native to the department of Meta (Colombian Orinoquia) and its adaptation process has allowed it to live in extreme tropical environments. The aim of this cross-sectional and descriptive study was to present the current state of the knowledge of asymmetries in some cephalic characters of the SM creole bovine. A total of 94 animals were studied (18 uncastrated males and 76 females) from three different farms, with an age range of 0.5–10 years. For each animal, two measurements of the ear (width and length) and two measurements of the horn (perimeter and length) were obtained in vivo. The degree of asymmetry was calculated as (R − L)/(R + L). Bilateral differences pointed towards a fluctuating asymmetry (e.g., a random variation in the trait that is expected to be perfectly symmetrical) biased towards right for ear width and horn perimeter, and towards left for ear and horn length. Since the development of these structures—ears and horns—is under the control of the same set of genes, the fluctuating asymmetry could constitute a reflection of a normal condition.

## 1. Introduction

Most biological structures present bilateral symmetry [1]. However, in the process of natural development, small disturbances or errors occur that have cumulative effects independently on both sides, causing an asymmetry of the structure [2]. Asymmetry can occur due to the influence of genetic and environmental factors [2,3]. The study of asymmetry has many theoretical and practical applications in conservation biology, quantitative genetics, evolutionary biology, medicine, and agriculture, among others [3,4]. Deviations from perfect symmetry can be measured as variances (or related measures of dispersion) of linear dimensions, variation in shape involving reference points, or continuous symmetry measures [5,6].

Asymmetry is defined as the deviation of a whole organism or a part of it from perfect symmetry. Basically, three types of asymmetries can be distinguished: fluctuating asymmetry, directional asymmetry, and antisymmetry [7,8].

Fluctuating asymmetry is the random variation in a trait (or characteristic) that is expected to be perfectly symmetrical on average; it is interpreted as an expression of the instability of development at the population level [7,9,10,11]. Developmental instability is the inability of an organism to resist the effects of perturbations during development [12] and is widely considered an indicator of environmental and genetic stress [4,9,13,14]. In response to environmental stress, errors occur in the development mechanisms of the biological structure, increasing developmental instability [15].

Directional asymmetry occurs when one side of the biological structure shows greater development than the other [6,9,16]. Antisymmetry is expressed as significant variations in asymmetry, but randomly on one side or the other, leading to a bimodal distribution of differences between the right and left forms of the morphological characteristic [6,11]. 

It is important to reiterate that, although developmental stability and asymmetry are individual characteristics, such patterns of bilateral variation can only be defined in statistical terms within a population context. In contrast to directional asymmetry and antisymmetry, fluctuating asymmetry does not favor one side; instead, it involves random differences between the right and left sides of a bilateral trait or measurement [4,7]. However, there is still a lack of knowledge about the diversity of approaches for the study of fluctuating asymmetry [5]. In our opinion, paired tests, which are used to compare two population means, can be effective statistical approaches to the study of paired asymmetries.

Contrary to fluctuating asymmetry, studies of directional asymmetry and antisymmetry have not been associated with stress and are characterized by a normal distribution with a mean value other than zero (directional asymmetry), and by a non-normal distribution with a mean value equal to zero (antisymmetry), respectively [4,17].

Some authors have stated that fluctuating asymmetry usually has a low heritable component [18,19], while the other two types of asymmetries may have an important genetic component [7,19]. Therefore, traits that do not require strict symmetry to function correctly may be strong candidates in which to use fluctuating asymmetry as an indicator of stress. Research in domestic animals has been published showing that fluctuating asymmetry occurs due to environmental or genetic stress. Mentions have been made with regard to metapodial asymmetry (metatarsus and metacarpus) in draft cattle [20], in hooves of thoroughbred horses [21], in secondary sexual characteristics in roosters [13], in fattening rabbits [22], in chicken fattening [23], in laying birds [17], in forelimbs in horses [24], in creole horse skulls [25], and in canine skulls [11], among others.

In the department of Meta, in Colombia’s Orinoquia, there is the SM creole bovine, which originated from the bullfighting bovine introduced by the Spanish in the 15th century. Since its introduction to Latin America, it has undergone a prolonged process of natural selection that has allowed it to develop adaptive characteristics (e.g., fertility) to live in extreme climatic environments (high temperature and relative humidity) and feed on fibrous forages. The bovine is defined as mesoline, eumetric, and orthoid. They are typically red in color, but some are black, brown, or isabelline. The ears are small and rounded. Males have crown-shaped horns, while females have lyre-shaped horns, which they use as defensive weapons [26,27].

Asymmetry in biological structures has attracted a substantial amount of research over the past three decades, especially in the area of evolutionary biology [28]. In the SM creole bovine, to date and to the authors’ knowledge, there is no published research on developmental instability, so this study has a specific interest in this bovine, as well as a general interest in other bovines. Given that the study of asymmetry is essential to determine small disturbances in the development process, we hypothesize that stress is the cause of the possible existence of asymmetries in the cephalic characteristics of the SM creole bovine. Therefore, the aim of this cross-sectional and descriptive study was to present the current state of the knowledge of asymmetries in some cephalic characters of the SM creole bovine.

## 2. Materials and Methods

### 2.1. Study Area

The current study was carried out in the department of Meta in Colombian Orinoquia (latitude: 1°36′29″ N and 4°54′24″ N; longitude: 71°4′42″ W and 74°54′9″ W). The region belongs to a zone of humid tropical forest and very humid tropical forest, with flat and undulating topography, and an altitude range of 200 to 450 m. In the rainy period (April to November), the relative humidity is 87% and the average ambient temperature is 26 °C; in the dry period (December to March), the relative humidity is 55%. The annual precipitation varies between 2700 and 3500 mm. The soils are acidic with high Al contents and mineral deficiencies (mainly P, Cu, Zn) [27]. 

### 2.2. Animals

In the present study, 18 uncastrated male and 76 female (n = 94) SM creole bovines were measured, with an age range of 0.5 to 10 years. Livestock producers sell the males at the time of weaning, so it was difficult to measure them, at least on the farms visited, hence the small number of males.

The animals came from three different farms located in the department of Meta (Colombia). The farms where the animals were measured belong to farmers associated with the Association of Creole and Colombian Cattle Breeders of the Eastern Plains (with ASOCRIOLLANOS being its acronym in Spanish). The farms were chosen by convenience after a meeting with the livestock breeders association. The farms were chosen based on the availability of infrastructure to measure the animals (handling “corral” and “brete”), road access, and permission from producers to measure the animals. The farms have the same management and maintenance conditions. Direct mating with several bulls is used on all farms [29]. The main activity is breeding; however, on some farms, milking is carried out manually with the calf present. The animals live on large areas of land where forage is scarce and water is distant. The main sources of food for the animals are native grasses used in grazing: gramalote grass (*Paspalum fasciculatum*), comino grass (*Homolepis aturensis*), hairy grass (*Trachypogon vestitus*), guaratara grass (*Axonopus purpussi*), maciega grass (*Paspalum virgatum*), lambedora grass (*Leersia hexandra*), black grass (*Paspalum plicatulum*), carretera grass (*Paratheria prostrata*), and introduced grasses (*Urochloa* spp.), among others. The feed is also complemented with mineralized salt [27]. 

### 2.3. Cephalic Measurements

For each animal, two measures of the ear (width and length) and two measures of the horn (perimeter and length) were obtained. Measurements were made with a tape measure on both sides, right (R) and left (L). Age was obtained based on the information available in the record of each animal. Ear and horn measurements were taken using standard morphometric methods [30].

- Horn perimeter (HP): Measurement around the base of the crown.
- Horn length (HL): Distance from the base of the crown to the apex of the horn.
- Ear length (EL): Distance from the base of the ear insertion to the vertex.
- Ear width (EW): Distance from the midpoint of the cranial border to the midpoint of the caudal border.

**Figure 1:**
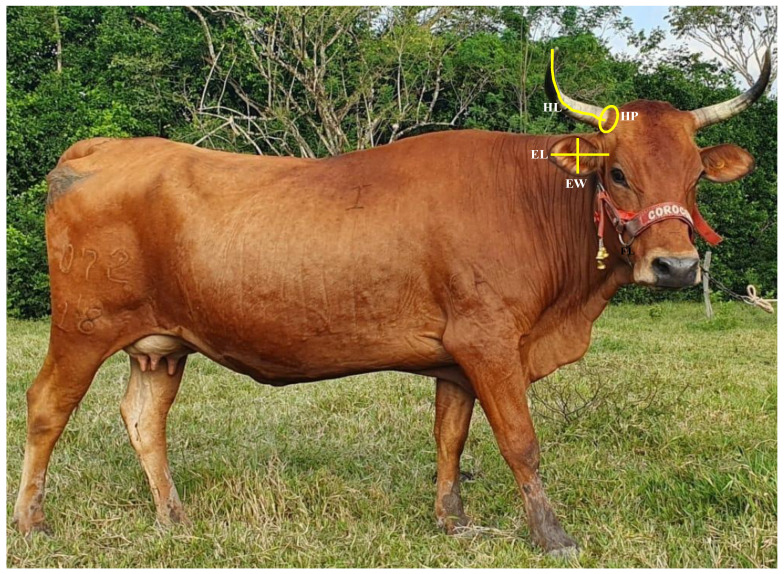
Graphic representation, with arrows, of the cephalic measures taken in the Sanmartinero creole bovine. HP = horn perimeter; HL = horn length; EL = ear length; EW = ear width. Photograph taken by the owner of the Punta Hermosa farm.

The measurements were taken directly from the animal using a measuring tape (Ovny, Inalmet, CO) graduated in centimeters. The animals were immobilized in a “brete” with a cement floor to facilitate taking measurements. All measurements were carried out by two trained students who participated in the data collection in the farm. One student took the measurement (in duplicate) with the tape measure and the other student wrote down the value of the measurement. Figure 1 shows a graphic representation of the measurements taken. 

### 2.4. Statistical Analysis

The degree of asymmetry was calculated as [(R − L)/(R + L)] for each variable. This relative index seems to us to be a more appropriate value than the mere variance of the difference between the right and left side and other alternative indices proposed [31], since this index eliminates the significant dependence on the mean of the character. The normality of the degrees of asymmetry was tested using a Shapiro–Wilk W test. A confidence level of 5% was used in all cases. The data were analyzed with the PAST v.2.17c statistical software [32].

## 3. Results

Table 1 shows the main simple statistics for the studied variables in the SM creole bovines.

Table 2 shows the degrees of relative asymmetry for the variables of ears and horns, which do not present a normal distribution in any of the cases, but the median is around 0, with marked skewness levels. Therefore, fluctuating asymmetry is suspected, biased towards right for ear width and horn perimeter, and towards left for ear and horn length.

## 4. Discussion

Environments exposed to constant climate changes and anthropogenic pressure can produce negative impacts that cause chronic stress in animals, affecting their development and even leading to extinction [14,33]. During development, the two sides of a biological structure share the same environmental and genetic conditions [34]; however, deviations can be caused by many stressors [9,31].

In the present study, for the length and width of the ears and the horn perimeter of the SM creole bovines, the bilateral differences point towards a fluctuating asymmetry, biased towards right for ear width and horn perimeter, and towards left for ear and horn length. Since the development of these structures—ears and horns—is under the control of the same set of genes [3,4,14], the fluctuating asymmetry could constitute a reflection of a normal condition. It has been reported that fluctuating asymmetry may be associated with damage to the horns [35]; however, in this study, during the collection of information, no damage was observed in the horns of the animals.

From the point of view of population biology, fluctuating asymmetry is important because it reflects the state of the adaptation and co-adaptation of a population [36]. In the case of the SM creole bovine, despite being adapted to the extreme environmental conditions of the Colombian Orinoquia region [26], the climatic changes that are currently being observed (e.g., rain in dry periods, sudden changes in temperature) may be factors that are influencing the normal development of cephalic characters (horns and ears). 

The study of horn size in ungulates has diverse scope for evolutionary and conservation biology [37]. Horn growth in bovids is indicative of habitat quality and population characteristics; however, little is known about the factors that affect it [38]. Horn growth is sensitive to changes in population density, climate, and the availability of food resources [6,39,40]. Ornamental characters (horns) are more susceptible to environmental stress than ordinary morphological characters because these are affected by directional sexual selection that would destabilize the genome [28,41]. Some studies have shown that population density increases horn asymmetry [42], while others mention that parasite load is associated with increased horn asymmetry [43]. The analysis of fluctuating asymmetry in biological structures is a promising line of research, not only to determine developmental instability, but also in the evaluation of animal behavior [35]. 

A limitation of the present study is the limited number of publications on the topic in creole bovines (asymmetry in horns and ears), which makes it difficult to delve into the discussion and make comparisons with other breeds. Another limitation is the small number of males, the reason for which was explained in the Materials and Methods section. However, we consider our results to be of interest. We suggest that further research related to asymmetries be conducted to detect possible developmental instability in lateralized characters (e.g., horns and ears) in Colombian creole bovines. These results serve as an example for future studies of other breeds of domestic ungulates.

## 5. Conclusions

Although the SM creole bovine is considered a resistant animal, it can be deduced that the environment in which they live causes stress in their development, and this may be responsible for the asymmetries detected. Climatic and other external factors must have a low impact on ear and horn structures at individual-level ontogeny; however, the authors are unaware of any study having been carried out in this sense. However, since the development of lateralized structures (ears and horns) is under the control of the same set of genes, the fluctuating asymmetry observed here could constitute a reflection of a normal condition. The ability to compare between breeds is limited as there are no studies on bovines, especially in terms of the characteristics studied in our research. This is the first study that has been carried out in a creole cattle breed. Our data will undoubtedly contribute in one way or another to the continued study of the Sanmartinero creole bovine and other bovine breeds. It is important to continue with research related to developmental instability in other characters in Colombian creole cattle.

## Figures and Tables

**Table 1:** Main simple statistics obtained from the biometry of the ear and horn of the SM creole bovines (n = 94 animals). Measures expressed in cm, except for the coefficient of variation, expressed in %. As expected, horn variables showed the highest variation, probably because they are linked to age.

Statistical	Ear Width		Ear Length		Horn Perimeter		Horn Length	
	R	L	R	L	R	L	R	L
Minimum	7.0	8.0	10.0	7.0	6.0	6.0	3.0	3.0
Maximum	13.0	14.0	23.0	23.0	30.0	31.0	42.0	51.0
Average	10.6	10.4	15.9	16.2	18.5	18.2	23.7	23.0
SD	1.04	0.95	2.01	2.23	4.15	4.06	8.99	9.59
CV (%)	9.78	9.09	12.57	13.72	22.36	22.29	37.93	41.66

R = right; L = left; SD = standard deviation; CV = coefficient of variation.

**Table 2:** Main simple statistics of the degrees of ear and horn relative asymmetries (relative differences between the right and left sides calculated as [(R − L)/(R + L)]) obtained from the SM creole bovines (n = 94). Skewness is a measure of the asymmetry of a distribution. A normal distribution exhibits zero skewness. Kurtosis indicates how many data reside in the tails. In other words, it represents how often outliers occur. The degree of asymmetry (skewness) and the outliers (kurtosis) of the distribution were not very high for all cases. *p*-value refers to Shapiro–Wilk test.

Statistical	Ear Length	Ear Width	Horn Perimeter	Horn Length
Minimum	−0.1	−0.4	−0.6	−0.2
Maximum	0.1	0.4	0.8	0.2
Average	0.0	0.0	0.0	0.0
SD	0.03	0.06	0.14	0.05
Median	0	0	0	0
Skewness	−0.14	0.27	1.69	−0.71
Kurtosis	0.53	28.99	17.90	1.56
Geometric mean	0.00	0.00	0.00	0.00
*W* of Shapiro–Wilk	0.952	0.529	0.564	0.910
*p*	0.00192	0.00000	0.00000	0.00001

SD = standard deviation.

## Data Availability

Data are available upon reasonable request to the second author.
